# Trends in Female First Authorship in Anatomical Science International: A 24-Year Analysis (2002-2025)

**DOI:** 10.7759/cureus.108488

**Published:** 2026-05-08

**Authors:** Takutoshi Inoue, Toru Yamamoto, Norio Kitagawa, Joe Iwanaga

**Affiliations:** 1 Department of Anatomy, Teikyo University School of Medicine, Tokyo, JPN; 2 Division of Dental Anesthesiology, Faculty of Dentistry and Graduate School of Medical and Dental Sciences, Niigata University, Niigata, JPN; 3 Department of Oral and Maxillofacial Anatomy, Graduate School of Medical and Dental Sciences, Institute of Science Tokyo, Tokyo, JPN; 4 Department of Neurosurgery, Tulane Center for Clinical Neurosciences, Tulane University School of Medicine, New Orleans, USA

**Keywords:** anatomy, authorship trends, female first authors, gender differences, women in science

## Abstract

In recent years, the promotion of gender equity and diversity in academic research has attracted increasing international attention. However, studies examining gender differences in authorship within the anatomical sciences remain limited. This study analyzed regional differences and temporal trends in the gender distribution of first authors in articles published in *Anatomical Science International*, the official English-language journal of the Japanese Association of Anatomists. All articles published between 2002 and 2025 were retrieved from PubMed, and the gender, country of affiliation, and article type of first authors were examined. Between-group comparisons were performed using the Bonferroni method, and temporal trends were assessed using the Cochran-Armitage trend test. Among the 1,029 articles included, 283 (27.5%) had female first authors. The proportion of female first authors significantly increased from 29/144 (20.1%) in 2002-2006 to 74/236 (31.4%) in 2022-2025. Regionally, Japan had the lowest proportion (99/487, 20.3%), compared with Asia excluding Japan (52/131, 39.7%) and other regions (134/411, 32.6%). By article type, the proportion of female first authors was lowest in review articles (34/182, 18.7%). These findings suggest that structural and contextual factors, including publication practices and access to research resources, may influence differences in female authorship. Promoting inclusive participation and re-evaluating academic structures may support further development in anatomical research.

## Introduction

Gender equality is a state in which all individuals, regardless of gender, share equal responsibilities, rights, and opportunities and can participate fully in decision-making processes [1]. This principle underpins the notion that women and men should enjoy equal rights and opportunities and is articulated in Goal 5 of the Sustainable Development Goals [2]. In recent years, the promotion of diversity within scientific research has gained global attention, with gender equality recognized as an essential component for the sustainable advancement of academic fields [3]. Although the proportion of female physicians has been increasing worldwide, women remain underrepresented in senior and leadership positions within the medical profession [4]. Similarly, in the field of anatomical science, the proportion of female researchers who hold senior roles or serve as principal investigators remains low, underscoring the need for policies and strategies to address persistent gender disparities [1,5,6]. Authorship patterns, particularly first authorship, are often considered indicators of academic participation and early-career research involvement and thus provide a useful proxy for examining gender disparities in research activity. Understanding authorship trends may also provide insights into the academic training environment and research participation in anatomical education.

*Anatomical Science International* (ASI), the official English-language journal of the Japanese Society of Anatomists, was launched in 2002 and serves as an international platform for disseminating anatomical research. However, no studies have systematically examined gender differences or regional disparities in first authorship among publications in ASI.

Therefore, this study aimed to analyze the gender, country of affiliation, and article type of first authors of papers published in ASI from 2002 to 2025. By doing so, we sought to clarify regional differences and temporal trends in the proportion of female first authors and to evaluate the publication patterns of Japanese female researchers in a Japan-based international anatomical journal.

## Materials and methods

This study examined articles published in ASI from 2002 (Volume 77, Issue 1) to 2025 (Volume 100, Issue 4). Bibliographic data for these articles were retrieved from PubMed on October 17, 2025, and subsequently organized and managed using Microsoft 365® Excel (Microsoft Corp., Redmond, WA, USA). As this was a retrospective study utilizing publicly accessible data, ethical review by our institution was waived.

Data collection

The paper title, publication year, and first author’s name were extracted from the compiled database. For each article, the first author’s gender (as determined in this study), country of affiliation, and paper type were individually verified and recorded in Microsoft 365® Excel.

To determine the gender of first authors, we conducted manual searches of the affiliated institutions’ official websites, researcher-oriented social networking services (e.g., ResearchGate and Researchmap), and general social media platforms (e.g., Facebook and Instagram). When gender could not be conclusively determined, it was inferred from the author’s name and publicly available photographs, drawing on general knowledge and contextual information.

Countries of affiliation were categorized into three regional groups: “Japan,” “Asia excluding Japan,” and “Other.” Paper types were classified into “Original Article,” “Review Article,” and “Case Report,” and only these article types were included in the analysis. Articles that did not represent original research, scholarly review, or case-based reports, such as editorials, letters to the editor, commentaries, corrigenda, errata, guidelines, short communications, and other non-research formats, were excluded. However, the Special Review Based on a Presentation made at the 16th International Congress of the International Federation of Associations of Anatomists (IFAA), published in 2004 (Volume 79, Issue 4), and the Original Paper published in 2005 (Volume 80, Issue 1) were reclassified as “Review Articles” upon detailed assessment of their content.

Using the finalized dataset, we analyzed gender differences across regions and paper types and further assessed five-year trends over the study period.

Statistical analysis

Between-group comparisons of categorical variables were performed using Fisher’s exact test. For pairwise comparisons between groups, p-values were adjusted using the Bonferroni correction to account for multiple testing. A two-sided p-value of <0.05 was considered statistically significant. All statistical analyses were performed using EZR (version 1.68; Saitama Medical Center, Jichi Medical University, Saitama, Japan) [7].

## Results

Number of publications

**Figure 1:**
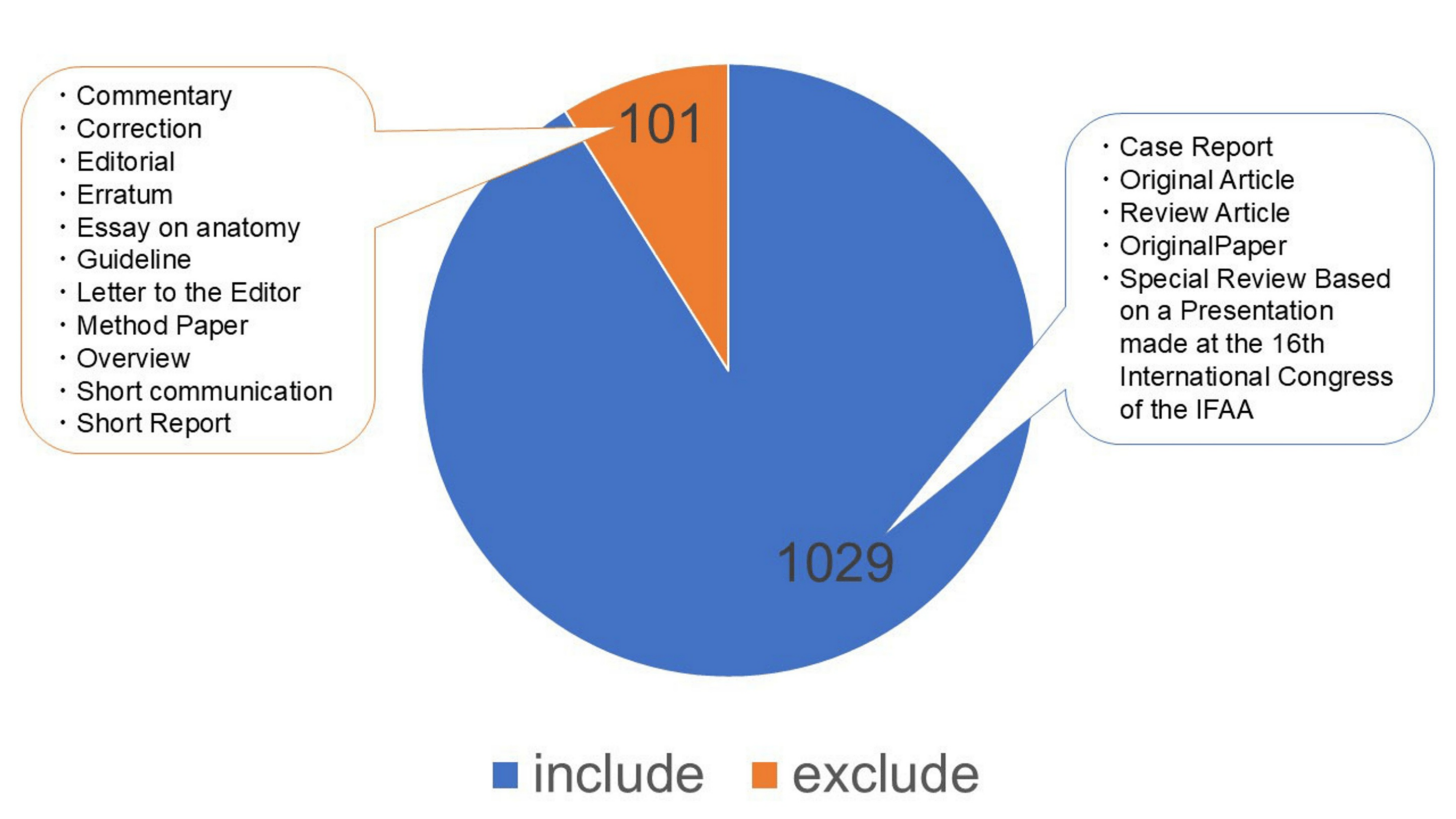
Number of publications in ASI (2002-2025) ASI: *Anatomical Science International*

A total of 1,130 articles were identified from the collected database. Of these, 1,029 articles that met the inclusion criteria and did not fall under the exclusion criteria were included in the final analysis (Figure 1).

Gender distribution

Of the 1,029 articles analyzed, 746 (72.5%) had male first authors, whereas 283 (27.5%) had female first authors (Table 1).

**Table 1:** Overall distribution of first-author gender in articles published in ASI (2002-2025) ASI: *Anatomical Science International*

Category	Total (n)	Male n (%)	Female n (%)
Overall	1029	746 (72.5)	283 (27.5)

Country of affiliation

The distribution of papers by the first author’s country of affiliation was as follows: 487 papers from Japan, 131 from Asia excluding Japan, and 411 from other regions (Table 2). Among these, the proportions of female first authors were 20.3% in Japan, 39.7% in Asia excluding Japan, and 32.6% in other regions, with Japan showing the lowest percentage. Within Asia, excluding Japan, India accounted for the largest number of papers, whereas Europe accounted for the largest share in the “other regions” category. Between-group comparisons revealed significant differences in the proportion of female first authors between Japan and Asia excluding Japan and between Japan and other regions (p < 0.001). In contrast, no significant difference was observed between Asia excluding Japan and other regions (Table 3).

**Table 2:** Distribution of first-author gender by country of affiliation in articles published in ASI (2002-2025) ASI: *Anatomical Science International*

Region	Total (n)	Male n (%)	Female n (%)
Japan	487	388 (79.7)	99 (20.3)
Asia (excl. Japan)	131	79 (60.3)	52 (39.7)
Other regions	411	277 (67.4)	134 (32.6)

**Table 3:** Pairwise comparisons of first-author gender distribution by region Statistical comparisons were performed using Fisher’s exact test. P-values were adjusted using the Bonferroni correction for multiple comparisons.

Comparison	Statistical test	p-value
Japan vs Asia (excluding Japan)	Fisher’s exact test	0.00014
Japan vs other regions	Fisher’s exact test	0.00011
Asia (excluding Japan) vs other regions	Fisher’s exact test	0.73

Paper type

The distribution of papers by article type was as follows: 665 original articles, 182 review articles, and 182 case reports (Table 4). In all categories, the proportion of male first authors exceeded that of female first authors. Between-group comparisons demonstrated a significant difference in the proportion of female first authors between original articles and review articles (p < 0.05). In contrast, no significant differences were observed among the other pairwise comparisons (Table 5).

**Table 4:** Distribution of first-author gender by article type in articles published in ASI (2002-2025) ASI: *Anatomical Science International*

Article type	Total (n)	Male n (%)	Female n (%)
Original article	665	465 (69.9)	200 (30.1)
Review article	182	148 (81.3)	34 (18.7)
Case report	182	133 (73.1)	49 (26.9)

**Table 5:** Pairwise comparisons of first-author gender distribution by article type Statistical comparisons were performed using Fisher’s exact test. P-values were adjusted using the Bonferroni correction for multiple comparisons.

Comparison	Statistical test	p-value
Original article vs case report	Fisher’s exact test	1
Review article vs case report	Fisher’s exact test	0.24
Review article vs original article	Fisher’s exact test	0.006

Trends in the number of publications and female authorship ratio presented in five-year intervals

**Figure 2:**
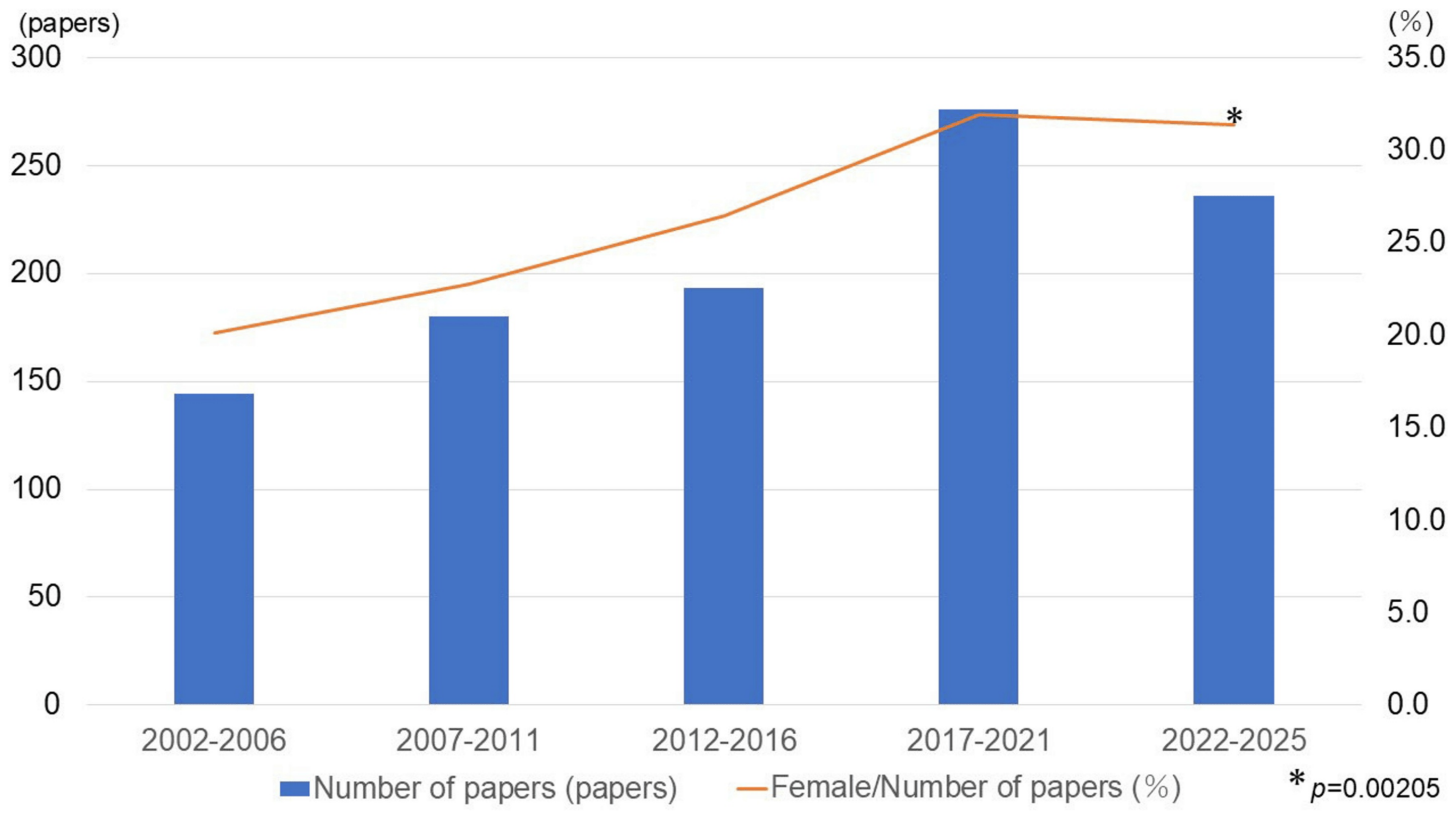
Trends in the proportion of female first authors in ASI (2002-2025) The proportion of female first authors was analyzed in five-year intervals. The p-value from the Cochran-Armitage trend test indicates a significant increasing trend over time. ASI: *Anatomical Science International*

When the proportion of female first authors was analyzed in five-year intervals, the Cochran-Armitage trend test demonstrated a significant upward trend over time (p < 0.05) (Figure 2).

Trends in first author country affiliation and female authorship ratio presented in five-year intervals

**Figure 3:**
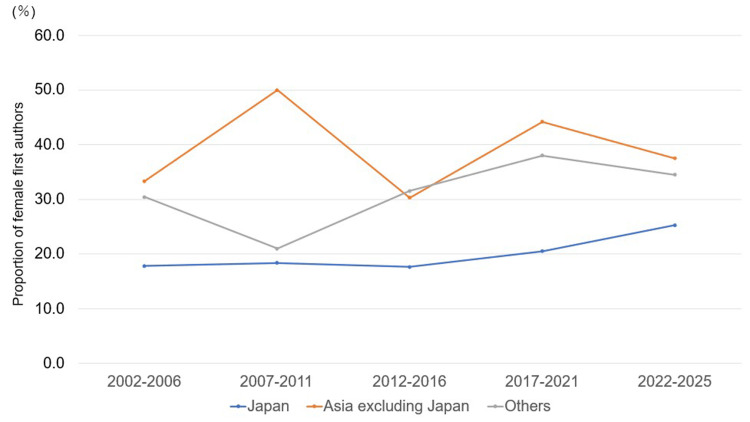
Trends in the proportion of female first authors by country of affiliation in ASI (2002-2025, n = 1,029) The proportion of female first authors was analyzed in five-year intervals by country of affiliation. The number of articles included in each interval was as follows: Japan, n = 118, 98, 68, 112, and 91; Asia excluding Japan, n = 3, 20, 33, 43, and 32; and other regions, n = 23, 62, 92, 121, and 113 for 2002-2006, 2007-2011, 2012-2016, 2017-2021, and 2022-2025, respectively. ASI: *Anatomical Science International*

When the proportion of female first authors was analyzed in five-year intervals by country of affiliation, Japan showed a gradual upward trend. In contrast, regions outside Japan maintained relatively high proportions but showed no clear further increases (Figure 3).

Trends in the types of publications and female authorship ratio presented in five-year intervals

**Figure 4:**
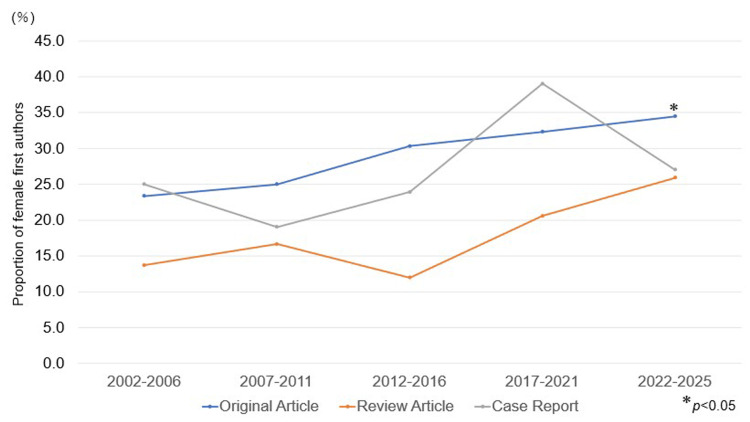
Trends in the proportion of female first authors by article type in ASI (2002-2025, n = 1,029) The proportion of female first authors was analyzed in five-year intervals by article type (original articles, review articles, and case reports). The number of articles included in each interval was as follows: original articles, n = 77, 120, 122, 201, and 145; review articles, n = 51, 18, 25, 34, and 54; and case reports, n = 16, 42, 46, 41, and 37 for 2002-2006, 2007-2011, 2012-2016, 2017-2021, and 2022-2025, respectively. The p-values represent the results of the Cochran-Armitage trend test, indicating changes in the proportion of female first authors over time for each article type.

When the proportion of female first authors was analyzed in five-year intervals by article type, the Cochran-Armitage trend test showed a significant upward trend in original articles (p < 0.05) (Figure 4).

## Discussion

This study analyzed the proportion of female first authors in original articles, review articles, and case reports published in ASI over the past 24 years. Among the 1,029 articles included, 27.5% had female first authors. The proportion of female first authors increased significantly from 20.1% in 2002-2006 to 31.4% in 2022-2025, demonstrating a significant upward trend over time. This finding is consistent with global reports indicating an increase in the proportion of women in the medical profession [4]. A previous analysis of five major anatomical journals between 2004 and 2023 also documented relatively stable female first authorship rates of 31-35% [6], and the recent proportions observed in our study (31.9% in 2017-2021 and 31.4% in 2022-2025) align with these international trends.

In contrast, clinical disciplines exhibit markedly lower proportions of female first authors. Previous studies have reported rates of 6.5-10.6% in orthopedic surgery journals [8,9] and 19.0% in Japanese cardiology journals [10], both of which are lower than those observed in the present study. These disparities may reflect social and institutional factors, including limited representation of women in senior positions, challenges in achieving work-life balance, and structural barriers within clinical environments [8].

Five-year interval analyses revealed a gradual increase in Japan, rising from 17.8% in 2002-2006 to 25.3% in 2022-2025. This trend may reflect the gradual increase in the number of female researchers and female graduate students in Japan.

With respect to country of affiliation, Japan exhibited the lowest proportion of female first authors (20.3%), compared with Asia excluding Japan (39.7%) and other regions (32.6%). Similar regional disparities have been reported in orthopedic surgery journals [8], Japanese cardiovascular journals [10], and international analyses of general internal medicine journals, in which Japan ranked among the lowest [11]. The Japanese proportion in our study is nearly identical to the 19.0% reported for Japan by Sebo et al. [11], suggesting a persistent lag in academic participation among women researchers in Japan. A five-year interval analysis further demonstrated a gradual increase in Japan, from 17.8% in 2002-2006 to 25.3% in 2022-2025, likely reflecting the increasing number of female researchers and graduate students.

Regarding article type, the proportions of female first authors were 30.1% for original articles, 18.7% for review articles, and 26.9% for case reports. The increasing trend observed in original articles is consistent with global reports indicating a rise in the number of female graduate students and researchers [12]. Case reports may be more accessible to individual researchers because they are generally less resource-intensive and can be conducted independently, thereby facilitating authorship opportunities for women.

In contrast, the relatively low proportion of female first authors in review articles should be interpreted in the context of structural characteristics of academic publishing. In ASI, review articles are generally commissioned by editors, and similar invitation-based practices are common across many academic journals. Such invitation systems and editorial networks tend to concentrate opportunities among established or senior researchers, who are disproportionately male [13]. A comparable pattern has been reported in ophthalmology journals [13], supporting the interpretation that structural mechanisms in scholarly publishing contribute to gender disparities in authorship.

Furthermore, discipline-specific structural factors inherent to anatomical sciences should be considered. Anatomy is a highly resource-dependent field that requires access to cadavers and advanced laboratory facilities, and the availability of such resources plays a critical role in shaping research activity. In this context, research opportunities often depend on established laboratory environments and supervisory networks, which may influence both entry into the field and career progression. Additionally, the challenges of securing competitive research funding and independent principal investigator positions may exacerbate disparities in access to research resources, ultimately affecting opportunities for academic dissemination. These findings may also reflect broader challenges in academic career development and access to research opportunities.

Additionally, the low proportion of female first authors in review articles in Japan may be influenced not only by institutional factors but also by academic and cultural contexts. In Japan, the practice of actively writing and submitting review articles may be less prevalent compared with other regions. The interaction between such cultural tendencies and invitation-based publication systems may further limit female researchers' opportunities to contribute to review articles.

Taken together, the observed differences in female authorship should not be interpreted solely as an individual-level issue but rather as a phenomenon shaped by the interaction of structural factors in academic publishing, access to research resources, and broader academic culture [14]. Similar patterns of persistent gender disparities in authorship have been reported across a wide range of medical disciplines, supporting the broader generalizability of the present findings [15]. This study has several limitations. First, it did not directly assess gender equality because data on submission rates and the underlying researcher population were unavailable. As a retrospective analysis using publicly available databases, the information was limited to what was reported in the published articles. In some cases, gender was inferred from publicly accessible information, and absolute accuracy cannot be ensured. Second, the country of affiliation was classified based on the first author's institutional address; thus, international collaborative work or multiple affiliations may not accurately reflect the authors’ regional backgrounds. Additionally, this study did not distinguish between international students and foreign faculty members working at Japanese institutions, which may have influenced the categorization. Third, because the analysis was limited to ASI, the findings may not be generalizable to other anatomical journals or related disciplines. This restriction to a single journal may also introduce selection bias, and first authorship was used as a proxy for academic participation, which may not fully reflect broader research contributions. Finally, various social and structural factors, such as childcare support, workplace conditions, promotion systems, invitation-based publication practices, and editorial board composition, may influence trends in female authorship. Still, these factors were not examined in this study.

Therefore, these interpretations should be considered speculative, as structural and cultural factors were not directly measured in this study.

## Conclusions

This study analyzed the gender, country of affiliation, and article type of first authors in articles published in ASI over the past 24 years. The overall proportion of female first authors was 27.5%, demonstrating a significant increase over time. However, differences were observed by geographic region and article type, with Japan consistently showing a lower proportion of female first authors than other regions.

In particular, although the proportion of female first authors increased in original articles, review articles remained predominantly authored by men, suggesting the influence of structural factors in academic publishing, including invitation-based publication practices. These findings indicate that observed differences in female authorship are not merely an individual-level phenomenon but are likely shaped by a combination of structural factors in academic publishing, access to research resources, and broader regional and cultural contexts.

Future efforts should focus on promoting inclusive collaboration that integrates diverse perspectives and on facilitating the participation of researchers from varied backgrounds, while also re-evaluating existing academic structures. Such approaches are expected to contribute to further advancement in anatomical research.
